# Borderline personality disorder and early psychosis: a narrative review

**DOI:** 10.1186/s12991-023-00475-w

**Published:** 2023-11-02

**Authors:** Arianna Biancalani, Lorenzo Pelizza, Marco Menchetti

**Affiliations:** Department of Biomedical and Neuromotor Sciences, Alma Mater Studiorum, Università Di Bologna, Via Zamboni 33, 40106 Bologna, BO Italy

**Keywords:** Borderline personality disorder, First episode psychosis, Schizophrenia spectrum disorder, Early psychosis, Early intervention, Early detection, Psychopathology, Diagnosis

## Abstract

**Background:**

The purpose of the present review was to summarize the main literature contribution on the relationship between borderline personality disorder (BPD) and early psychosis. While retracing the historical path of the term “borderline”, specific attention was paid to psychotic and psychotic-like symptoms in BPD. Its relationship with At Risk Mental State was evaluated, as well.

**Methods:**

This search was conducted on PUBMED/MEDLINE and PsycInfo, looking for “Borderline personality disorder, First Episode Psychosis, Early Psychosis, Ultra-High Risk AND/OR Clinical High Risk” for psychosis.

**Results:**

Eight pertinent papers were identified on this topic. Their main findings were then discussed. The term “borderline” has undergone different changes in meaning and use, despite always referring to states considered on the fence between neurosis and psychosis. However, considering the history of psychopathology and its relationship with diagnostic manuals, little attention has been given to its psychotic features. Being those symptoms highly burdensome, this neglect has often led to misdiagnosis and under-treatment.

**Conclusions:**

Psychotic symptoms in BPD can be severe and distressing. Nonetheless they can be easily neglected, and when found they challenge clinicians in defining a differential diagnosis to distinguish between BPD and Psychosis Spectrum Disorders. Given specific needs and interventions for these different conditions, a dimensional, rather than categorical, approach should be considered, as well as specific care pathways and monitoring should be advised.

## History of the concept of “Borderline” disorder

For over a century, since it was first used as a psychoanalytic concept by Stern in 1938 [1], the term “*borderline*” has experienced a continuous change in use and understanding. In this respect, Stern originally used the word to describe a cluster of patients who were not likely to respond well to psychoanalytic therapy and that he believed was somehow different both from schizophrenia and neuroses [2]. In 1952, Knight [3] was the first to define a “borderline state”, which was conceptually very close to schizophrenia, but also had neurotic features and identified a temporary “ego state” that the patients could enter and exit, thus being affected only for a given time. This idea interestingly resembles a previous one by Zilboorg [4], a psychoanalyst who at first described patients with “ambulatory schizophrenia”, in which it was reported one of the two main roots of the “borderline” concept: i.e., its psychotic features (shared with schizophrenic disorders).

It was in 1967, then, that the term predominantly ceased to identify a mild form of schizophrenia, when Kernberg [5] borrowed it to describe one of the possible levels of personality organizations (namely, “psychotic”, “neurotic” and “borderline”). Quite differently from what Stern had stated, Kernberg used it to qualify a disorder not likely to change during time and closer to our understanding, as well [6].

In the first edition of “A Glossary of Psychoanalytic terms and Concepts” [7], Moore and Fine defined “borderline” as “a descriptive term referring to a group of conditions which manifest both neurotic and psychotic phenomena without fitting unequivocally into either diagnostic category”. It is probably because of this indefiniteness that, despite the many attempts to classify it, it has always remained vague and hardly harmonized in the psychiatric field.

After a long journey through different schools of thought, where a “Borderline Syndrome” [8] and a “Borderline Disorder” [9] were described, in 1979 Spitzer and Endicott theorized the belonging of the “borderline patient” (as it was commonly perceived) either to a Schizotypal Personality Disorder or to an Unstable Personality Disorder [10]. Indeed, even if the term “borderline” had been misused for decades, there was no official description in any previous diagnostic manual. These two terms referred to the two main uses of the word “borderline”, identifying, on one hand, a group of patients more closely related to schizophrenia, and a group of patients predominantly characterized by “unstable affect, interpersonal relationships, job functioning, and sense of identity” [11]. Later, the task force who worked at the third edition of the Diagnostic and Statistical Manual of mental disorders (DSM-III), replaced the term “unstable personality disorder” with “borderline personality disorder”, which was clinically much more usual and wasn’t subject to misinterpretation on the actual stability of personality disorders. Despite Spitzer and Endicott [10] remarked how not mutually exclusive the two diagnoses were, but dimensionally likely to be integrated, psychotic-like features formally ceased to be associated with Borderline Personality Disorder (BPD) (for a brief summary of the history of BPD concept, see Table 1) [12–15].

**Table 1:** Brief summary of the history of BPD conceptualizations

Author(s)	Quotes
Bleuler (1911) [12]	“If we examine some individuals more closely, we often tend to suspect the presence of simple schizophrenia without, however, being able to make a definite diagnosis at the given time; but very often, after days or years, our suspicions can be confirmed. Thus, there is no doubt that many simple schizophrenics are at large whose symptoms are not sufficiently pronounced to permit the recognition of mental disorder. If one observes the relatives of our patients, one often finds in them peculiarities which are qualitatively identical with those of the patients themselves, so that the disease appears to be only a quantitative increase of the anomalies seen in the parents and siblings. Such mild cases are often considered to be “nervous” or “degenerated” individuals, etc. But if we follow the anamnesis of those who are admitted to the hospital in later years because of an exacerbation of their difficulties, a criminal charge, a pathological drinking bout or some such episode, we can usually find throughout the entire past history of the individual mildly pathological symptoms which in the light of their recent illness unquestionably have to be considered as schizophrenic. There is also a latent schizophrenia, and I am convinced that this is the most frequent form, although admittedly these people hardly ever come for treatment. It is not necessary to give a detailed description of the various manifestations of latent schizophrenia. In this form, we can see in mice all the symptoms and all the combinations of symptoms which are present in the manifest types of the disease. Irritable, odd, moody, withdrawn or exaggeratedly punctual people arouse, among other things, the suspicion of being schizophrenic. Often one discovers a concealed catatonic or paranoid symptom and exacerbations occurring in later life demonstrate that every form of this disease may take a latent course”
Freud (1913) [13]	“Often enough, when one sees a case of neurosis with hysterical or obsessional symptoms, mild in character and of short duration, a doubt which must not be overlooked arises as to whether the case may not be one of insipient dementia praecox and may not sooner or later develop well-marked signs of this disease”
Stern (1938) [14]	“It is well known that a large group of patients fit frankly neither into the psychotic nor into the psychoneurotic group, and that this border line group of patients is extremely difficult to handle effectively by any psychotherapeutic method”
Zilboorg (1941) [4]	“The less advanced cases have been noted, but not seriously considered. When of recent years such cases engaged the attention of the clinician, they were usually approached with the euphemistic labels of borderline cases, incipient schizophrenias, schizoid personalities, mixed manic-depressive psychoses, schizoid manics or psychopathic personalities”
Hoch and Polatin (1949) [15]	“Some psychiatrists concede that the clinical and psychodynamic structure of these cases differs from the neuroses –although retaining a great deal of resemblance to the neurotic disturbances–and call them "borderline cases. …Again, others are struck by the similarity of the mental changes and personality structure to schizophrenia and will diagnose them as schizophrenics. The writers would like to emphasize that this group of patients is not small”
Knight (1953) [3]	“Patients with borderline states were falling apart on the couch”
Kernberg (1967) [5]	“The ego pathology differs from that found in the neuroses and the less severe characterological illnesses on the one hand, and the psychoses on the other. These patients must be considered to occupy a borderline area between neurosis and psychosis. The term borderline personality organization, rather than “borderline states” or other terms, more accurately describes these patients who do have a specific, stable, pathological personality organization; their personality organization is not a transitory state fluctuating between neurosis and psychosis”
Moore and Fine (1968) [7]	“A descriptive term referring to a group of conditions which manifest both neurotic and psychotic phenomena without fitting unequivocally into either diagnostic category”
Grinker and co-workers (1968) [8]	“This book contains the first reported results of a lengthy research program on hospitalized borderline patients whose ego-functions were studied through multiple observations on their daily behaviors. In general, the informal diagnostic term of borderline as well as several synonyms in our nosological classification has long been used without standard definition as a convenient term with which to label cases of clinical unclarity. In this first systematic investigation of the phenomena clinically observed for at least several decades as borderline, we have attempted to understand what the term really denotes, define its characteristics, and determine whether it encompasses subgroups or categories”
Gunderson and Singer (1975) [9]	“This review of the descriptive literature on borderline patients indicates that accounts of such patients vary depending upon who is describing them, in what context, how the samples are selected, and what data are collected. The authors identify six features that provide a rational means for diagnosing borderline patients during an initial interview: the presence of intense affect, usually depressive or hostile; a history of impulsive behavior; a certain social adaptiveness; brief psychotic experiences; loose thinking in unstructured situations; and relationships that vacillate between transient superficiality and intense dependency. Reliable identification of these patients will permit better treatment planning and clinical research”
Spitzer and co-workers (1979) [11]	“Although there is a large psychiatric literature on various "borderline" conditions, there has been no agreement as to the definition of the concept. A review of the literature reviewed two major uses of the term: Borderline Schizophrenia and Borderline Personality. Two item sets were developed to provide diagnostic criteria for the two concepts. High sensitivity and specificity were demonstrated for both item sets using data describing 808 borderline and 808 control patients. These criteria will be used in the forthcoming DSM-III classification for the categories of Borderline Personality Disorder and Schizotypal Personality Disorder”

*BPD* borderline personality disorder

## Defining psychotic symptoms in BPD

According to the fifth edition of the Diagnostic and Statistical Manual of mental disorders (DSM-5), BPD is described by nine different criteria [16]. Notably, the ninth point mentions “transient, stress-related paranoid ideation or severe dissociative symptoms”, which somehow recalls the historical ambivalence of BPD psychopathology, considered on the fence between psychotic and neurotic symptoms [17]. However, psychotic features have not always been this relevant. It was only in 1994, when the DSM-IV was published, that BPD was acknowledged again a reference to potential psychotic experiences, after being strictly set apart from the schizotypal personality disorder in DSM-III [18].

For the first time in the history of The International Classification of Diseases and Related Health Problems, the 11^th^ revision (ICD-11) includes a description of psychotic-like symptoms “in situations of high affective arousal”, when identifying a borderline pattern for personality disorders [19].

Even if what should or should not be included in the description of BPD could seem only a theoretical issue, it is evident how the accuracy of definitions and criteria can affect the quality of diagnosis [20]. Also, even if the debate around the appropriateness of considering psychotic aspects a core diagnostic feature is still open, it is unarguable that these symptoms can be serious for patients who experience them [21]. What is needed, then, is an effective effort to address patients’ needs and to improve specific treatments, accordingly.

## Evaluating the burden: the data so far

Psychotic symptoms in BPD are often underrated and considered temporary or mainly associated with stress [17]. Yet, going deeper into the study of their phenomenology and comparing them to the “proper” psychotic features of schizophrenia, similarities and differences depict a very specific pattern of characteristics.

On one hand, psychotic symptoms in BPD seem to be phenomenologically very similar to those experienced by patients with psychosis spectrum disorders [17, 22]. As a direct consequence, differential diagnosis can be a challenge, thus leading to potential fluctuations in diagnosis and to mistreatment [23]. Also, despite the usual underestimation of psychotic experience in BPD, these symptoms can cause an extremely high burden. Among the most distressing psychotic symptoms in BPD, auditory verbal hallucinations (AVH) play a central role [21] and up to 50% of patients report them [24]. Indeed, some of the most worrying data suggest that AVH in BPD are associated with increased suicidal ideation, suicide attempts and hospitalizations [18, 22, 25]. If compared to those in schizophrenia, AVH in BPD have not been found to differ in frequency, duration, location, loudness, or conviction [17]. Coherently, misdiagnosis mostly happens when AVH differ from the common understanding of the diagnostic manuals and either meet criteria for “First Rank Symptoms” (FRS), and are perceived as coming from outside the head or when of lasting duration [20].

On the other hand, psychotic symptoms differ in some ways between BPD and Psychosis Spectrum Disorders. First, delusions, conceptual disorganization and negative symptoms seem not to be as common as in schizophrenia [17]. When describing AVH, patients with BPD refer a greater distress and negativity in content, yet they seem to be able to manage them better than patients with schizophrenia can, where commentary voices are also more frequent [17]. Given that AVH usually appear earlier in BPD (mean age at onset = 16 years) [22] and that these patients show better resistance, early diagnosis becomes fundamental in preventing the worsening of the symptoms and in aiming at the best possible quality of life.

Interestingly, a recent study [22] compared psychotic symptoms in adolescents with a full-criteria diagnosis of BPD to those experienced by subthreshold BPD patients. The main differences, concerning psychotic symptoms, psychoticism and occupancy, were observed between the full-threshold group and the subthreshold group. Despite the awareness that psychotic symptoms are always a risk factor for worse functioning, regardless of the diagnosis, this finding suggests that a diagnosis of BPD should be a hint for careful management and monitoring.

After defining the significance of psychotic symptoms in BPD, questions around treatment and management arise. There are many reasons why there is a high need for studies to assess the benefit of psychotherapy and antipsychotics in these specific cases [22]. One of the most relevant is the understanding that neurocognitive impairment is known to be greater in BPD with psychotic features [26]. One of the hypotheses is that such impairment can compromise mentalization, which consequently makes social cognition weaker and psychotic symptoms (starting with paranoid phenomena) more likely to arise. The use of antipsychotic drugs, which a recent review [27] reported to be effective in these patients, should also be furtherly discussed. As analyzed in a review by Beatson [20], the most studied antipsychotics for AVH in BPD are olanzapine (2.5–10 mg daily), aripiprazole (2.5–10 mg daily) and quetiapine (50–150 mg daily). Additional results about the efficacy of aripiprazole compared to placebo on AVH in young patients (aged 15 to 25 years old) are awaited soon, since a RCT on the topic is on its way to be published [28].

## BPD in at-risk mental states and first-episode psychoses

Given the challenge of differentiating BPD with psychotic features from psychosis spectrum disorders, it is evident that this becomes even more difficult when subtle, subthreshold psychotic symptoms are involved (Table 2). Indeed, since transient psychotic symptoms can be present in both BPD and early psychosis, there is a significant overlap between “At-Risk Mental States” (ARMS) and BPD spectrum psychopathology with attenuated psychotic features at presentation [23].

**Table 2:** Main empirical findings on BPD psychopathology in patients with early psychosis

Gleeson (2011) [29]	Treating co-occurring first-episode psychosis and borderline personality: a pilot randomized controlled trial “Results: The results showed that it was feasible to recruit and assess a high risk and complex group of patients who were agreeable to study participation. Specialist first-episode treatment plus specialist early intervention for borderline personality was an acceptable and safe treatment.” (Gleeson et al. 2012)
Schultze-Lutter (2012) [30]	Personality disorders and accentuations in at-risk persons with and without conversion to first-episode psychosis: personality disorders and psychosis risk “Conclusions: Unexpectedly, schizotypal PD was infrequent and did not predict conversion. Conversion was best predicted by schizoid PA, indicating more severe, persistent social deficits already at baseline in later converters. This corresponds to premorbid social deficits reported for genetic high-risk children and low social functioning in at-risk patients later converting to psychosis. Further, PDs occurred frequently in at-risk patients irrespective of conversion. As psychopathology and personality relate closely to one another, this result highlights that, beyond the current narrow focus on schizotypal PD, personality-related factors should be considered more widely in the prevention of psychosis.” (Schultze-Lutter et al. 2012)
Ryan (2017) [31]	Borderline personality pathology in young people at ultra-high risk of developing a psychotic disorder: borderline personality pathology “Conclusions: Many UHR patients present with concurrent borderline personality features. The psychotic experiences reported by UHR patients with borderline personality features were not limited to paranoid ideation, supporting the idea that borderline personality disorder may include a wider range of psychotic symptoms than previously thought. It is further possible that the psychotic symptoms experienced in this group could also be indicative of an emerging psychotic disorder.” (Ryan et al. 2017)
Francey (2018) [32]	Does co-occurring borderline personality disorder influence acute phase treatment for first-episode psychosis? “Conclusion: Young people with co-occurring clinician-rated BPD and FEP experienced greater difficulty accessing standard care for FEP and received relatively different treatment, including different pharmacotherapy, compared with those FEP patients without BPD. There is a need to develop new clinical guidelines and effective treatments for this specific subgroup with early psychosis and co-occurring BPD that take into account interpersonal and "premorbid" aspects of their presenting problems.” (Francey et al. 2018)
Paust (2019) [33]	Borderline personality pathology in an at risk mental state sample “Results: We found a significant correlation between borderline symptomatology and positive symptoms assessed by the structured interview for prodromal symptoms. There were no associations between basic symptoms for psychosis and borderline symptoms. In addition, there was no influence of borderline symptomatology on the rate of transition into a manifest schizophrenic disease. Summary: In conclusion, borderline personality disorder should not be an exclusion criterion for the screening for psychosis or for an early intervention treatment. On the other hand, not every patient with borderline personality disorder, (especially those not suffering from hallucinations, unusual thought content, or persecutory ideas) should automatically be screened for the risk of developing a psychotic disorder.” (Paust et al. 2019)
Carrasco (2021) [26]	Persistent psychotic symptoms and neurocognitive deficits in borderline personality disorder “Neurocognitive impairment and its association with psychotic symptoms in BPD suggest that a substrate of impaired social cognition underlies emotional dysregulation and impulsive behaviors in these patients. In other words, the greater the social cognitive deficit, the higher is the possibility of primitive and paranoid phenomena, such as auditory hallucinations or delusional explanations. This probably associates with inability for mentalization in these patients, and hence, the need of specific psychotherapeutic interventions different of non-psychotic BPD.” (Carrasco et al. 2021)
Hayward (2021) [21]	A cross-sectional study of auditory verbal hallucinations experienced by people with a diagnosis of borderline personality disorder “Conclusion: The findings suggest that AVH is a legitimate and distressing symptom of BPD and a treatment priority for some patients. The relative independence of AVHs from other BPD symptoms and emotional states suggests that psychological treatment may need to be targeted specifically at the symptom of AVHs. This treatment could be adapted from cognitive behaviour therapy, the psychological intervention that is recommended for the treatment of AVHs in the context of psychosis.” (Hayward et al. 2022)
Schandrin (2022) [34]	Co-occurring first-episode psychosis and borderline personality pathology in an early intervention for psychosis cohort “Conclusion: BPP is a common occurrence in psychotic disorders and is associated with more severe hallucinations and depression with higher risks of self-harm. Specific interventions need to be developed.” (Schandrin et al. 2022)

*BPD* Borderline Personality Disorder

Starting from this background, the *aim* of this narrative review was to examine the main findings on BDP in patients with early psychosis reported in the literature to date.

### Methodology

The search was conducted on MEDLINE/PubMed and psycInfo, looking for “Borderline personality disorder, First Episode Psychosis, Early Psychosis, Ultra-High Risk AND/OR Clinical High Risk” for psychosis. We specifically analyzed papers written in English and published by May 31, 2023. We found 8 pertinent papers on this topic. Their main findings were reported and discussed (see Table 2 for details) [29–34].

## Results

Clinical similarity on such psychotic features can be possibly explained going deeper into the study of psychopathology. According to a study by Zandersen and Parnas [35], most patients with BPD (considering different stages of illness) meet criteria for a schizophrenia spectrum disorder (which includes Schizotypal Personality Disorder). This observation gives space to different thoughts. First, as it is known from a historical perspective, the choice to differentiate BPD from SPD has long been debated, since many patients often meet both criteria. All things considered, it is logical to assume that BPD as a concept ends up being over-inclusive [36]. This comes as a direct consequence of a methodological shift, which comprises the use of an atheoretical diagnostic manual, based on behaviors rather than personality structure and prototypes [35]. In addition, time should be taken to reflect upon the psychopathological concept of BPD and consider a step “back” to a theoretical understanding of the disorder, which was originally very close to schizophrenia. Specifically, there are some key BPD features, like “identity disturbance” and “feeling of emptiness”, which might resemble other symptoms, nonetheless belonging to the Schizophrenia Spectrum Disorders [35], like the so-called “disorders of the self”. Given the similarity, it would be helpful to build back awareness around the meaning DSM criteria have on a “narrative level” [35] and how deeply these concepts can be explored on a “core” psychopathological level. Differential diagnosis would then be easier. Also, this would allow the acknowledgment of those highly severe cases of BPD, who share a common psychopathological ground with schizophrenia. Interestingly, some effort has been made to determine whether different subgroups of BPD could explain its heterogeneity. Smits and co-workers [37], for instance, identified three clusters of BPD patients sharing common characteristics. Among these, a schizotypal/paranoid type was described as very close to SPD, with introjective and psychotic-like features, which inevitably posed questions around its risk for psychosis. This subgroup was not as represented as the Core BPD and the Extravert/Externalizing subtypes in the study, yet seemed to be more likely to present late at mental health services, thus being more vulnerable to negative outcomes.

So far, when examining ARMS, no predictive meaning towards transition to psychosis has been found in those patients who presented BPD symptoms [33]. Nonetheless, the severity of BPD psychopathology hasn’t been associated either with clinical higher or lower risk for psychosis, thus suggesting how every patient with BPD having attenuated psychotic symptoms and/or meeting cognitive-perceptive basic symptoms criteria (i.e., COGDIS and COPER) should be monitored, regardless of the scores [31, 33] (see also Table 2, for details on main empirical findings about BPD psychopathology in early psychosis). Then, this is even more important, since it is known that, while keeping in mind that not all people with BPD need to be screened for psychosis, their co-occurrence results in a worse functioning and response to treatment [38]. Indeed, a possibility of co-occurring diagnoses of BPD and schizophrenia spectrum disorder may also occur. In this respect, Bahorik and Eack [38] reported that at 1-year follow-up, patients with schizophrenia and comorbid BPD showed significantly less improvement in psychiatric symptomatology (particularly hostility and suspiciousness), as well as global functioning, and were re-hospitalized at significantly higher rates than individuals without BPD. The authors suggested that the co-occurrence of schizophrenia and BPD was not infrequent and that BPD had a significant negative longitudinal impact on the course and outcome of subjects with schizophrenia.

When it comes to analyzing the impact of BPD on First Episode Psychosis (FEP), recent findings suggest how the overlap of the two disorders can lead to an increased risk for depression and self-harm [34]. Surprisingly, despite a higher severity, this combination is not associated with a major number of hospitalizations. One possible explanation is that patients with BPD may undergo a substantial under-treatment, probably due to a common underestimation of their symptoms.

Considering that, according to Francey and colleagues [32], the subgroup of patients presenting with FEP and BPD can represent up to a quarter of the total cases of FEP, specific attention should be paid to the treatment. The same study reports how this cluster of patients is more likely to receive a lower dosage of antipsychotics. This finding underlies how specific guidelines would be helpful in avoiding a stigmatizing approach and consequent mistreatment.

## Discussion

BPD psychopathology can be found in patients presenting with early psychosis. Also, psychotic, or psychotic-like symptoms can be the main features already at the first contact of BPD patients with mental healthcare services. These statements lead to two main clinical considerations. First, personality structure and pathology should be explored in people presenting with early psychosis, since it is known personality disorders can hide specific needs and affect the response to treatment. This may also require developing new clinical guidelines and effective treatments for young patients with early psychosis and co-occurring BPD that take into account “premorbid” and interpersonal aspects of their presenting problems. Secondly, special attention should be paid to BPD patients who carry a high burden of disease, with intense psychological suffering and bad functioning. Indeed, the severity of symptoms and functional impairment should alert mental healthcare professionals and lead to a further investigation of basic symptoms and potential psychotic psychopathology (either attenuated or full-blown). In this respect, psychotic symptoms can be viewed as “trans-diagnostic phenomena” [39], with psychotic experiences in schizophrenia spectrum disorders and BPD sharing similarities, which raises the question of whether they are underlain by the same neural mechanism [40] and have common risk factors (such as previous traumatic events, family history of psychotic disorder, substance misuse) [41]. Taking this into account, theoretical questions around how BPD should be defined and perceived inevitably arise. Should BPD meeting psychotic symptoms or ARMS criteria be considered more severe and worthy a personalized approach? If so, should psychotic experiences be reconsidered as core diagnostic criteria? Since definitions do not always meet the reality of everyday clinical practice, what, anyhow, should be achieved is the understanding of the high suffering this kind of patients can go through. Moreover, the evidence that BPD patients with psychotic signs are often at higher risk of developing a wider range of negative outcomes (including suicidal thinking and behavior) cannot be ignored, especially when considering treatment and monitoring.

In conclusion, sometimes understanding this specific vulnerability requires mental health professionals to go beyond narrow diagnostic categories and embrace what is known as a “dimensional approach” to psychopathology and psychiatric disorders. Future studies on early psychosis and BPD should thus recognize both the dimensional and dynamic nature of psychopathological symptoms and evolving phenotypes across the transition from childhood to adulthood by adopting a clinical staging approach [42]. Such an approach needs to include the measurement of personality pathology, in order to focus on the etiological factors and treatment options for psychotic symptoms in BPD. Also, taking into account a “network approach to psychopathology” [43, 44], common risk factors, such as trauma and substance abuse, could be considered as possibly interacting with each other, thus concurring to overlapping psychopathological categories. This could be the first step in the complex path to better understand the relationship between psychosis spectrum disorders and psychotic experiences being found in severe personality disorders.

## Data Availability

Not applicable.
